# Simplified Treatment of Teeth with Exposed Pulps

**Published:** 1897-01

**Authors:** Withold Lindemann

**Affiliations:** Rybinsk, Russia.


ARTICLE IV.

### SIMPLIFIED TREATMENT OF TEETH WITH EXPOSED PULPS.

BY WITHOLD LINDEMANN, OF RYBINSK, RUSSIA.

Translated from the *Correspondens-Blatt fur Zahnarste.*

The pulp laid bare during excavation is covered with iodol cement, which serves as a simple capping; this can, however, only be accomplished in those cases in which the exposed part has only the circumference of a point. The slight hæmorrhage which occurs here almost regularly is stopped by means of carbolic acid solution, and any pain there may be is removed by the application of a small pellet of cocaine, or a strong solution of cocaine. Thereupon the neighboring carious parts are removed with the utmost care as far as this is possible.

For the present the cavity must not be dried out with hot air; but it is thoroughly syringed out several times with luke-warm water in order to get rid of the congealed blood. A phosphate cement which does not set too quickly is then mixed with iodol (cement and iodol-powder in equal parts) to a very soft consistency; a mixture thus prepared is very sticky and remains plastic for a considerable time.

The cavity is then rapidly and thoroughly dried with amadon, and a portion of the cement, the size of a hempseed, is placed upon the exposed pulp. This iodol cement should be applied very lightly by means of a smooth stopper moistened with oil; directly the patient feels pain, the pressure must at once cease. The cement may also be pressed home by means of a dry pledget of cotton-wool dipped in talc powder.

It is well only to close the cavity provisionally and not to insert the filling until the following day; the slight pains which are generally experienced during the setting of the cement will then gradually have passed away.

It will be observed that the cement has become sufficiently hard to protect the pulp against the pressure exercised during the insertion of the filling; the cement, however, is usually distributed too unequally in the cavity, therefore it has in part to be drilled out again—of course with great care, so as not to lay the pulp bare again.

As this mode of capping can be carried out without the employment of metal caps, it is especially suitable for all cases in which the small dimension of the cavity and also the approach to it do not permit of the application of a cap.

I have employed this method in many cases without meeting with any failures.

In places difficult of access, and also where pulpitis has appeared (even if of a slight character only), it is advisable not to attempt the capping of all, but merely to cauterize the pulp; for this purpose shreds of cobalt or arsenious paste may be used.

Kirk's arsenious paste (with a slight modification) one may prepare one's self, of an excellent quality, by extremely finely triturating in a porcelain mortar arsenious acid (two parts), which is difficult to pulverize, with one part of pumice and a little carbolic acid in such a manner that even within a 150 magnifying power no arsenious crystals can be distinguished; hereupon are triturated with the mass two parts of cocaine, and one part of menthol; other additions are superfluous. A very small quantity of this paste is laid upon a pellet of cotton-wool the size of a pin's head, and this plug is applied direct upon the exposed pulp. A temporary closure of the crown-cavity with sandarac or mastic solution, however, requires a little knack.

In order to avoid the inclosure of the paste, and prevention of its action by the resinous solution, the cotton wool soaked with the resin should never be laid direct upon the arsenious paste; the two plugs should always be kept separate by means of a pledget of cotton-wool moistened with water. The action of the arsenic will then be undisturbed, and the hardened resin will not exercise any pressure upon the pulp.

At the expiration of twenty-four hours the pulp in most cases will be so void of sensibility that it can be partly removed; otherwise the cauterization should be repeated.

For the removal of the pulp, I do not in such cases employ nerve extractors, but bore out the crown pulp by means of an aseptic engine-bur, and also partly the pulp of the root. It is not necessary for this purpose to introduce the bur deeper than from two to three mm. into the root-canal. The debris caused by the bur is removed by means of powerful syringing with warm water, whereupon the cavity is carefully prepared for the reception of the filling. After a thorough drying out of the cavity a piece of soft charcoal, the size of a hemp-seed, impregnated with oleum cassiæ, is laid in the pulp chamber, and pressed into the root-canals with a good sized plugger in such a manner that it comes to lie upon the pulp-remains which are still sensitive; should the space permit, one can place a second piece of the charcoal in thus prepared. The oil of cassia which is expressed is then removed, together with any traces of blood which may be present, by means of cotton-wool, and the cavity is hermetically closed with cement or plaster of Paris. Plaster of ordinary consistency is prepared and held in readiness upon a glass-slab; of this a small quantity is laid upon the charcoal which is in the cavity. Should the plaster not adhere to the oily layer of charcoal, it is pressed tight by means of a dry plug of cotton-wood dipped in talc-powder; it will then adhere to the tooth-substance, and cover the charcoal. If the latter is insufficiently covered, a little more plaster is added, and condensed with cotton-wool. One must then wait for the plaster to harden before inserting the filling.

For the covering of the charcoal C. Ash & Sons' chloride of zinc cement (Superior) is the most suitable of all the cements known to me. The phosphate cements, which in a plastic condition do not possess any affinity for water, are more difficult to use, and cannot well be fixed on the floor of the cavity. Nor can the chloride of zinc cement by itself be fixed with the aid of pluggers; on the other hand, it can be introduced with ease if the cement, mixed moderately soft, is pressed down with cotton-wool plugs dipped in talc powder.

The sealing of the cavity with cement is certainly much more reliable than with plaster of Paris. After the hardening (the cement will harden under water), the crown cavity is carefully prepared for the introduction of the filling. All undercuts, etc., and also the cervical margin, are cleared of plaster, or, rather, of incompletely hardened cement, and every trace of superfluous material is removed. The cofferin is then applied, the cavity dried, and filled in any way desired.

Of 243 teeth, which I have filled during the last two years by this method, I had to record in the early part of this time only four failures, which, however, are to be accounted for by insufficient cauterization and defective cleansing of the pulp cavity; yet even in these cases the pains (pulpitis) which supervened disappeared on the repeated application of arsenic. Periostitis did not, however, occur in a single case. I prefer, therefore, this method to any other, and have discarded definitely the filling of the roots after the cauterization of the pulp.

With pulpless teeth this method cannot be adopted; in the treatment of these teeth the antiseptic filling of the entire root-canal cannot perhaps be avoided.

Discoloration of the dentine is not to be feared by the employment of this method, if due care has been taken to cover the charcoal at the transparent dentine layers.—*Dominion Dental Journal.*

